# Tilt-less 3-D electron imaging and reconstruction of complex curvilinear structures

**DOI:** 10.1038/s41598-017-07537-6

**Published:** 2017-09-06

**Authors:** Emad Oveisi, Antoine Letouzey, Duncan T. L. Alexander, Quentin Jeangros, Robin Schäublin, Guillaume Lucas, Pascal Fua, Cécile Hébert

**Affiliations:** 1 Interdisciplinary Centre for Electron Microscopy, École Polytechnique Fédérale de Lausanne (EPFL), CH-1015 Lausanne, Switzerland; 2 Electron Spectrometry and Microscopy Laboratory, École Polytechnique Fédérale de Lausanne (EPFL), CH-1015 Lausanne, Switzerland; 3 Computer Vision Laboratory, École Polytechnique Fédérale de Lausanne (EPFL), CH-1015 Lausanne, Switzerland; 4 Scientific Centre for Optical and Electron Microscopy, Swiss Federal Institute of Technology in Zurich (ETHZ), CH-8093 Zurich, Switzerland

## Abstract

The ability to obtain three-dimensional (3-D) information about morphologies of nanostructures elucidates many interesting properties of materials in both physical and biological sciences. Here we demonstrate a novel method in scanning transmission electron microscopy (STEM) that gives a fast and reliable assessment of the 3-D configuration of curvilinear nano﻿structures, all without needing to tilt the sample through an arc. Using one-dimensional crystalline defects known as dislocations as a prototypical example of a complex curvilinear object, we demonstrate their 3-D reconstruction two orders of magnitude faster than by standard tilt-arc TEM tomographic techniques, from data recorded by selecting different ray paths of the convergent STEM probe. Due to its speed and immunity to problems associated with a tilt arc, the tilt-less 3-D imaging offers important advantages for investigations of radiation-sensitive, polycrystalline, or magnetic materials. Further, by using a segmented detector, the total electron dose is reduced to a single STEM raster scan acquisition; our tilt-less approach will therefore open new avenues for real-time 3-D electron imaging of dynamic processes.

## Introduction

Accurate insights into the physical and chemical properties of nanoscale materials and devices are critical for controlling their functionality^1^. Transmission electron microscopy (TEM) is a versatile technique that provides such information for a broad range of disciplines. However, being a 2-D projection image, a single TEM micrograph is generally insufficient to identify the 3-D morphology of nanostructures: information pivotal to understanding complex 3-D structural nature^2–5^.

In principle, tomographic or stereoscopic techniques can be used to reconstruct the 3-D nature of a sample from a series of 2-D projection images acquired across an angular range or arc^6–10^. While in an X-ray CAT (computer-aided tomography or computed axial tomography) scanner this is done by keeping the object fixed and rotating the source and detectors around the object, in standard TEM tomography the source and detectors are fixed and the object is rotated, typically acquiring a sequence of projected images of the same properties at small angular intervals over a relatively large tilt arc (typically >100 images over ±60–70**°** angular range)^11^. Associated with this methodology are a number of challenges. Firstly, images used to make a tomogram should fulfil the projection requirement: i.e. they must exhibit an intensity that varies monotonically with respect to the properties to reconstruct^12^. As will be seen for imaging dislocations, this is particularly hard to achieve, and so prone to artefacts, for imaging conditions that depend on diffraction contrast^13–16^. A precise alignment of the diffraction condition through a large tilt arc usually involves the use of sample holders specifically designed for such experiments^17^. Secondly, the mechanical tilt of the specimen typically results in some lateral movement between images in one tilt arc series. Removing these movements by image alignment is often problematic. Thirdly, the total electron dose during acquisition of so many images can, in many cases, lead to structural changes in a sample during the data acquisition, thus resulting in a corrupted data set^18, 19^.

Aberration-corrected STEM optical sectioning techniques has been successfully applied to identify the 3-D distribution of isolated dopant atoms^20–23^ and the configuration of dislocations at the atomic scale^24^, through recording a depth/focal series instead of a tilt-series. However, while, for optical sectioning techniques, data can be acquired more rapidly and with more precise image registration than with tilt-series tomography, its resolution for more general cases remains a big issue^25, 26^. A severe elongation effect and artefacts due to the missing-cone problem currently limit the application of depth-sectioning techniques to imaging isolated point-like/1D/2D objects such as single atoms, on-axis atomic columns and atomic planes^25, 27^. For objects of other shapes, it is very challenging to obtain a directly interpretable 3-D reconstruction without image processing that applies prior knowledge of the specimen^25, 27^. A solution has been proposed to solve this, through the use of incoherent scanning confocal electron microscopy (SCEM), however it depends on a complicated experimental implementation^28^. Moreover, all these techniques involve acquisition of an image series and thus the prolonged exposure to electrons remains an issue. A combination of uncorrected SCEM and sample tilt pair imaging has been used for depth measurements by parallax, but was limited to linear structures with a rather coarse depth discrepancy^29^.

Here, we present a STEM-based technique where, instead of rotating the object, we effectively rotate the source and detector. Further, by taking advantage of a sophisticated, proprietary image processing algorithm, we reduce the data input required for 3-D reconstruction to just two images taken at different incident beam angles (effectively a tilt-less “stereoscopic pair”), thereby increasing the efficiency of data acquisition by one to two orders of magnitude compared to standard TEM tomography techniques.

Currently, our novel and rapid method is applicable to the 3-D reconstruction of complex curvilinear nano﻿﻿structures. Here, we demonstrate the method using crystal dislocations. These 1-D structural defects are used both because of their challenges for tomographic imaging (in particular the problem of maintaining a constant deviation from the Bragg diffraction condition for different tilts), and because of their importance to society in governing the structural and opto-electronic properties of many significant engineering and semiconductor materials^30–32^. Experimentally, our method requires only a standard modern FEG-equipped scanning TEM instrument (STEM) and standard specimen holder (dual axis for diffraction contrast imaging). The tilt-less imaging exploits the convergent illumination of the STEM electron probe to create different virtual incident beam tilts while maintaining a constant image contrast (and diffraction condition) over the stereoscopic image pair. Further, unlike conventional TEM (CTEM) imaging, in STEM imaging the transmitted electrons do not need to be refocused to form an image, but simply need to be detected^33^. As a result, while CTEM images of thick specimens, which are often needed for identifying the 3-D path of dislocations, are subject to blurring associated with the chromatic aberration of inelastically-scattered transmitted electrons by the post-specimen imaging lens, the use of STEM inherently reduces this effect and so gives improved image contrast. In the next sections we describe the full principle of tilt-less 3-D electron imaging, and experimentally demonstrate and validate it using the challenging case of dislocations.

## Results

### Principle of the tilt-less 3-D electron imaging

**Figure 1:**
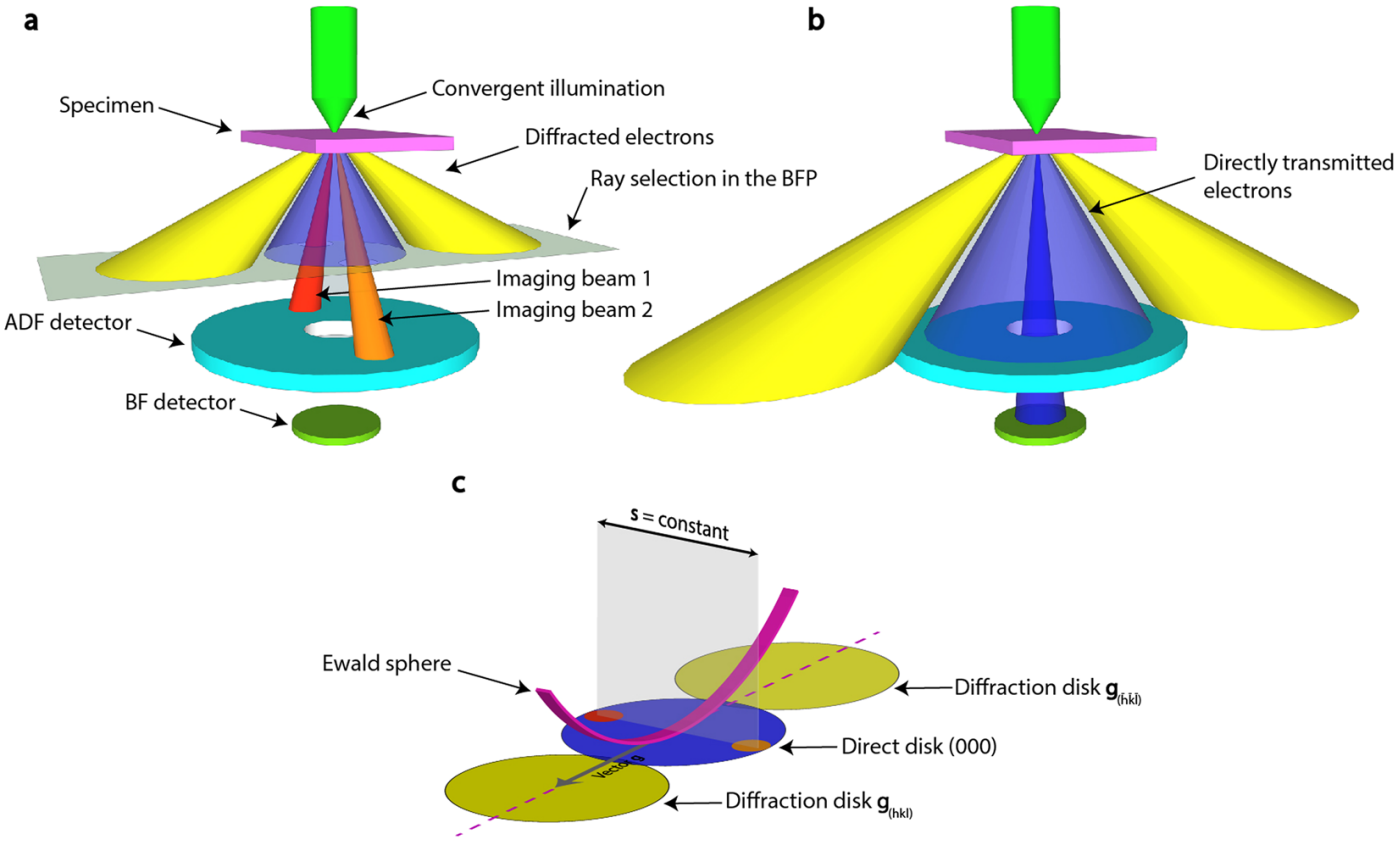
Schematics illustrating the tilt-less 3-D electron imaging technique. (**a**) Imaging with an inclined direct beam (imaging beam 1 and 2), coloured in red and orange: a BFP aperture is placed on the edge of the directly transmitted disk, which is collected by an annular detector. (**b**) Imaging with a conical beam along the optical axis, coloured in blue: the BFP aperture is removed, and the on-axis BF detector receives the centre of the direct disk. (**c**) Illustration of the convergent beam electron diffraction (CBED) pattern corresponding to the diffraction condition shown in (**a**). Orange and red circles indicate the position of the BFP aperture for imaging conditions of beam 1 and 2, respectively. Drawings are not to scale.

The microscope setup for the tilt-less 3-D imaging is schematically illustrated in Figure 1. When the TEM is operated in scanning mode, a focused convergent electron probe of less than a few Å in diameter and with a convergence angle of a few tens of mrad (determined by electron optical setup) is scanned in a raster pattern across the specimen^34, 35^. As the electron beam transmits through the specimen it undergoes coherent elastic scattering, resulting in the formation of a convergent-beam electron diffraction (CBED) pattern in the back focal plane (BFP) of the objective lens^34^. For each probe position, the integrated intensity of the CBED pattern illuminating a detector in the back focal (or conjugate) plane is displayed in its corresponding image pixel. If calibrated in scattering angle, the typical CBED pattern of a crystalline sample consists of electron diffraction disks with a diameter equal to the probe convergence angle 2α, with a centre-centre spacing ***g***_(*h k l*)_ (diffraction vector) from the direct (0 0 0) disk that corresponds to twice the relevant Bragg diffraction angle, *θ*_(*h k l*)_^36^. By considering the incident solid cone of illumination as consisting of parallel rays of different illumination angles, it can be seen that each position within the direct (0 0 0) disk essentially corresponds to a specific electron beam direction prior to the sample. Selecting and integrating different regions of the direct beam disc is therefore equivalent to tilting the parallel incident illumination in CTEM^37, 38^, thereby giving STEM images viewed from slightly different directions. We now use this principle to acquire bright-field (BF) stereo image pairs from the (0 0 0) disc. To acquire stereo images with the largest possible shift angle, the camera length is tailored so that the rim of the (0 0 0) disc lies on an annular STEM detector. By recording the signal on this detector, a pair of stereo BF images can be acquired with 2α angular difference (−α/+α of tilt from the optic axis, in the range of few tens of mrad). The two images are differentiated by inserting an aperture in the BFP to act as a physical mask before the STEM detector, i.e., using the “objective aperture” of CTEM, and sequentially recording images with first one side and then other selected by displacing the aperture appropriately (see Figure 1a). Alternatively, the use of a new-generation segmented annular STEM detector allows for simultaneous acquisition of stereo images even without displacing an aperture; a method with great promise for real-time 3-D imaging. Lastly, in addition to the stereo images of −α/+α effective tilt angle, a third BF image using a conical beam along the optical axis (0 degree tilt) can be recorded with the on-axis BF STEM detector, in order to assist the image reconstruction (Figure 1b).

In this setup, the effective tilt axis is normal to the direction between the two selected regions of the (0 0 0) disk. Unlike standard TEM tomography, it is therefore independent of the tilt axis of the microscope goniometer. This is of particular interest when diffraction contrast is exploited for image formation, because there is no need to align a particular diffraction condition over the tilt range. In any sort of Bragg diffraction condition (e.g. zone axis or two-beam cases), the sign and magnitude of the deviation from the Bragg condition (characterized by deviation parameter ***s***) changes along the diffraction vector ***g***_(*h k l*)_^39^. However, by instead acquiring the stereo images along an axis perpendicular to the diffraction vector ***g***_(*h k l*)_, ***s*** remains constant and hence the images share the same diffraction contrast; so fulfilling a fundamental criterion for 3-D reconstruction.

### Application to dislocation imaging and reconstruction

By combining this novel setup in STEM with a dedicated reconstruction algorithm, we now demonstrate the power of this technique in reconstructing the 3-D path of dislocation lines.

1-D crystal defects called dislocations play a fundamental role in governing the properties of many materials across a broad range of applications, from structural components in infrastructure and transportation, to transistors and optoelectronic light emitters^40^. Since the first observation of dislocations in the 1950s^41, 42^, which came long after their theoretical postulation^43, 44^, much research has been devoted to investigating dislocation interactions, such as the “dislocation starvation” mechanisms in mechanically deformed micro- and nano-pillars, or the threading dislocations that propagate in non-epitaxial GaN-based light-emitting devices^14, 31, 32, 45, 46^. In this regard, TEM has been a key tool for 2-D investigations of dislocations, both by way of diffraction contrast imaging with the so-called “***g∙b*** analysis” technique, or by high resolution techniques exploiting phase contrast^47, 48^. 3-D imaging and reconstruction affords new perspectives on dislocation geometry, for instance to unravel the complexity of their networks and interaction mechanisms^2, 5, 49, 50^. A fast and reliable 3-D dislocation analysis greatly enhances the possibilities of making such studies for scientific and technological advances. The tilt-less method presented here provides this capability, as we now demonstrate by measuring the 3-D arrangement of threading dislocations (TDs) in a heteroepitaxial gallium nitride (GaN) membrane; a key optoelectronic material in which the 3-D geometry of the as-grown defect network is critical to its properties^14^.

**Figure 2:**
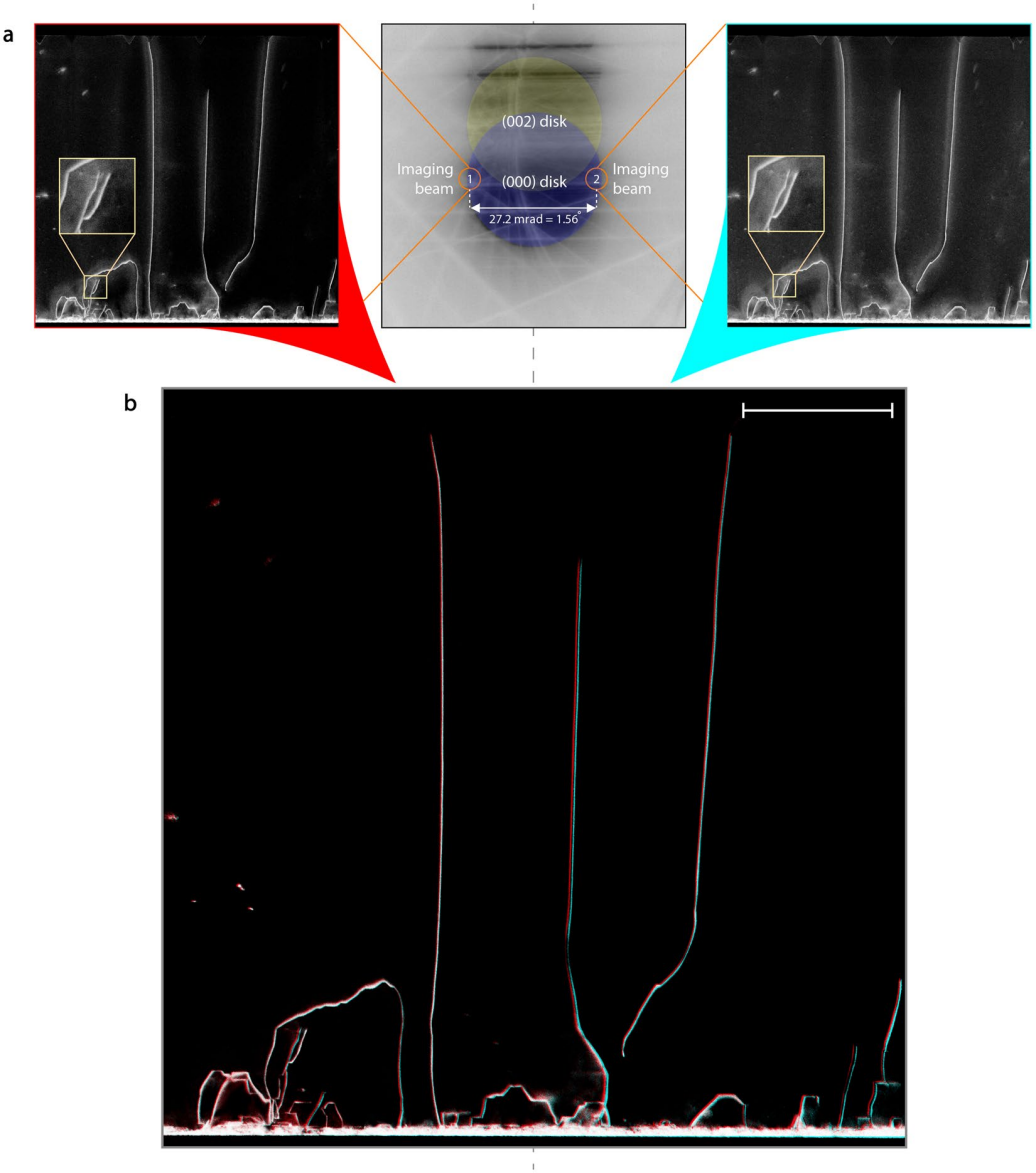
Tilt-less 3-D STEM imaging of dislocations in the GaN layer of a heteroeptaxial InAlN/GaN-based membrane. (**a**) Illustration of the stationary CBED pattern of the specimen set with positive deviation from a ***g***_(*h k l*)_ = (0 0 2) two-beam Bragg diffraction condition. The BF-STEM stereo micrographs (inverted contrast) with a virtual tilt angle of 27.2 mrad (1.56°) correspond to imaging beam 1 and 2 on the CBED pattern. (**b**) Anaglyph illustrating the 3-D configuration of dislocations, produced by combining the stereo micrographs of (**a**). The anaglyph must be viewed along the tilt axis shown by the dashed-line, with special coloured glasses (red glass for left eye and cyan glass for right eye). Scale bar, 500 nm.

Figure 2a shows the CBED pattern of the specimen (beam convergence angle 2α of 27.2 mrad) that has been mechanically tilted to excite a near two-beam condition with ***g***_(*h k l*)_ = (0 0 2) with a slightly positive deviation parameter ***s***, and the pair of stereo images recorded by choosing the two counterpart regions of the direct beam along a scattering axis perpendicular to ***g***_(*h k l*)_. Setting ***s*** > 0 helps to obtain a narrow dislocation line width in the image, and attenuates the oscillatory contrast of dislocations^51, 52^ to ensure a successful 3-D reconstruction^14, 53^.

**Figure 3:**
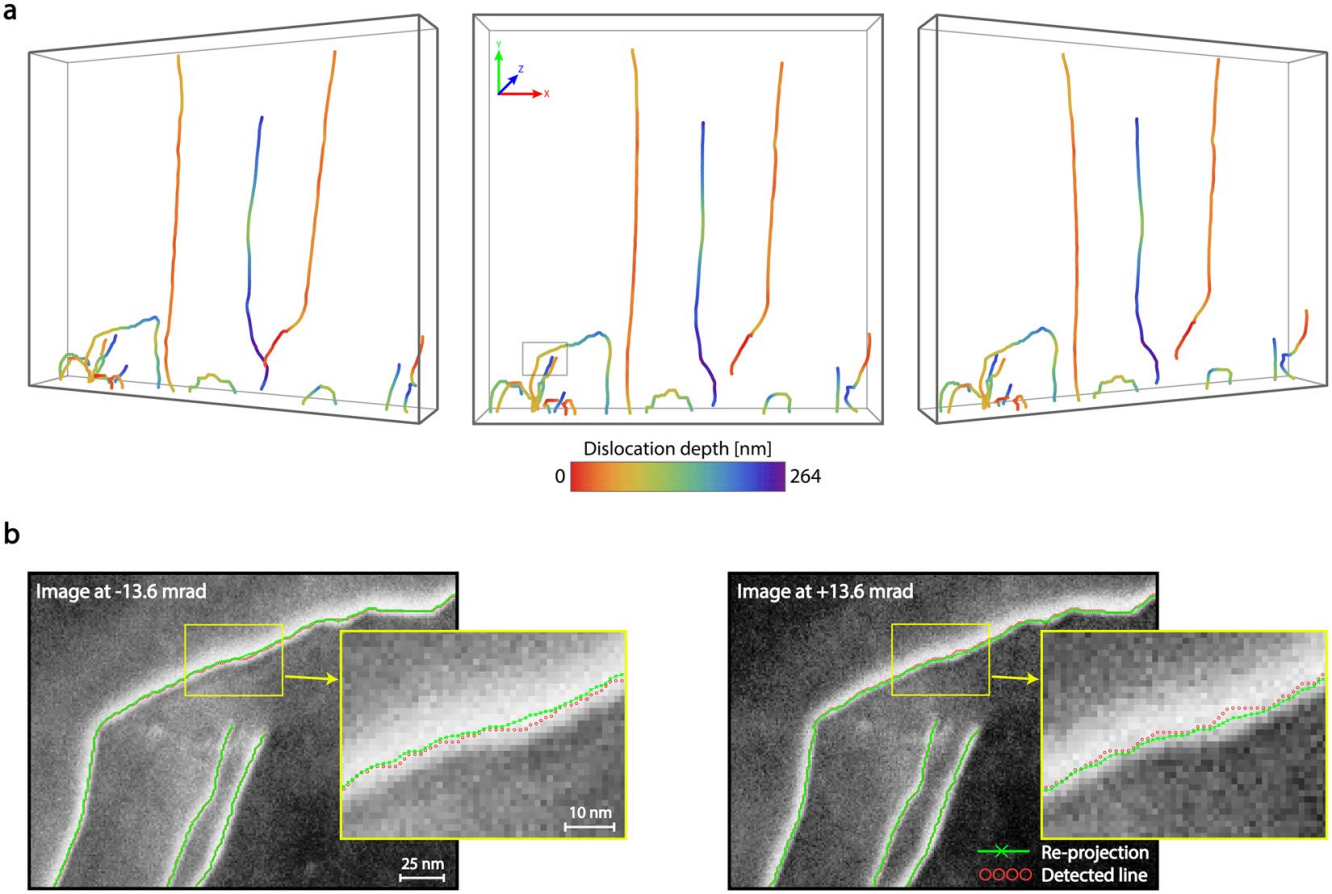
Tilt-less 3-D reconstruction of dislocation arrays of Figure 2. (**a**) Reconstructed dislocations are viewed from different perspectives. The colour code indicates the depth (*Z* direction) of each dislocation segment. The box size is 2458 × 2458 × 264 nm. (**b**) Re-projection of the reconstruction (green lines) into the corresponding tilt-less stereo images (of ±13.6 mrad virtual tilt) of a dislocation array marked by a square in (**a**). The red circles show the dislocation segments detected by the algorithm.

Comparing the pair of stereo images, whose effective viewing directions are separated by 27.2 mrad (1.56°), reveals significant differences in the projected paths of the dislocations (see Figure 2a), readily providing a sense of three-dimensionality as observed in the form of an anaglyph (Figure 2b). These differences are caused by viewing the dislocations’ varying depths in the thick, cross-sectional TEM lamella from different angles, and increase if a larger convergence angle (virtual tilt angle) is used (see Supplementary Figure S1). The resulting stereo micrographs are treated with a propriety algorithm based on standard stereoscopic triangulation techniques to recover their full 3-D geometry, even in spite of the very small angular shift between the images. To exploit this, we developed a 3-D reconstruction algorithm that employs well-established computer vision techniques to match contours across images and infer their 3-D shape^54^. The algorithm relies on so-called *active contours* ^55^, and initially extracts 2-D curvilinear structures from individual images using a semi-automated method that efficiently computes a path between given start and end points in the dislocation image^56^. In the case of a large dislocation density or dislocation entanglement, ambiguities can arise where their paths overlap in the images of the stereo pair. In this case, additional points can be manually introduced to assist the 2-D line detection process. Thereafter, the structures are matched across the images, thereby establishing correspondences between individual points. To this end, the algorithm extracts a sparse set of reliably matched points using cross-correlation as well as order constraints to perform piecewise linear interpolation, and then extends the matching to all individual points. Using the obtained correspondences, an algorithm based on gradient-descent is applied to all parameters to estimate the projection and rotation parameters for all images. Finally, given these parameters and the correspondences, the 3-D structure of the dislocations is reconstructed by triangulation (Figure 3a). The algorithm takes the specificity of the setup into account by introducing customized smoothing techniques based on Gaussian filters of different sizes to overcome the non-homogeneous nature of the noise along different axes. Thus, meaningful results are produced even with limited angular shift between views.

Inspection of the 3-D data set (see Figure 3a and Supplementary Movies) shows a step-movement of TDs during the growth of the first 200 nm of the film, changing the line direction along the $\langle 1\,\bar{2}\,1\,3\rangle$ and $\langle 1\,\bar{1}\,0\,0\rangle$ directions or bending by 90° towards coalescence boundaries. Through these mechanisms, many of *(* ***a*** + ***c*** *)*-type TDs change, for instance annihilating via loop formation or reacting to produce ***a***-type TDs as the film thickens^57, 58^. Such data, in which both the content of a particular dislocation’s character and its line direction are identified, are of considerable interest for understanding their origin and effect on light emitting device performance. This information can be correlated with the growth dynamics, in order to define predictive models for developing more effective TD reduction strategies and hence optimize the properties of the device^58, 59^.

### Fidelity assessment

Here we assess the accuracy of the tilt-less reconstruction: namely the fidelity with which the path of a curvilinear object is rendered. To gain a first insight, the accuracy is evaluated by comparing the estimated tilt between image pairs derived from the algorithm to the experimentally-measured probe convergence angle; the two values are within 5% of each other, lending support to the accuracy of the triangulation process. Because the reconstructed 3-D contours are smoothed, their re-projections into the images may not exactly match the original 2-D contours. However, the re-projected images stay within the width of dislocation contrast (Figure 3b) and the mean difference is 1.2 pixels, corresponding to only 1.4 nm along the *X*- and *Y*-axes. The reconstruction accuracy along the *Z*-axis (depth) is less easy to quantify. In order to estimate it, experiments are performed on simulated data based on a known object. These show that for stereo tilts beyond 1° the reconstruction given by the algorithm has an accuracy of a couple of pixels error or better (see Supplementary Figure S2). Further, the thickness of the specimen obtained from the tilt-less 3-D reconstruction (264 nm, defined as the difference of the dislocation’s exit points along the *Z*-axis) matches very well to the thickness obtained from electron energy-loss spectrometry measurements (242 nm ± 10%), as well as to that determined from the conventional tilt-based tomography (~253 nm) that we describe in the following. Overall, these results corroborate the fidelity of the tilt-less imaging technique and reconstruction when applied to well discriminated (i.e. well resolved) curvilinear objects.

### Comparison with conventional tomography

To benchmark our method further, we compare the path of the dislocations as identified by the tilt-less technique to their path as determined by conventional STEM tomography. For this comparison, a tilt-series from −25° to +25° at 1° increments was acquired and then reconstructed using the simultaneous iterative reconstruction technique (SIRT) (see Figures S3 and S4 and Suppleme﻿ntary Movies). To maintain a constant deviation parameter throughout the tilt range, the tilt-axis of the holder must lie parallel to the diffraction vector set for imaging. However, due to the imprecision of this alignment as well as the elastic anisotropy of this material, in this case diffraction contrast varies at large tilt angles. Note that a stronger misalignment leads to a non-linear variation in contrast between the images in the series, preventing a successful reconstruction^53, 60^. We emphasize that this limitation is removed in the tilt-less image acquisition, since the effective tilt axis can be chosen independently of that of microscope goniometer in order to obtain stereo images with the same diffraction contrast. This will be essential for analysing dislocation networks in specimens that cannot be specifically sectioned on favourable crystallographic orientations or that are polycrystalline with domains of different orientations.

**Figure 4:**
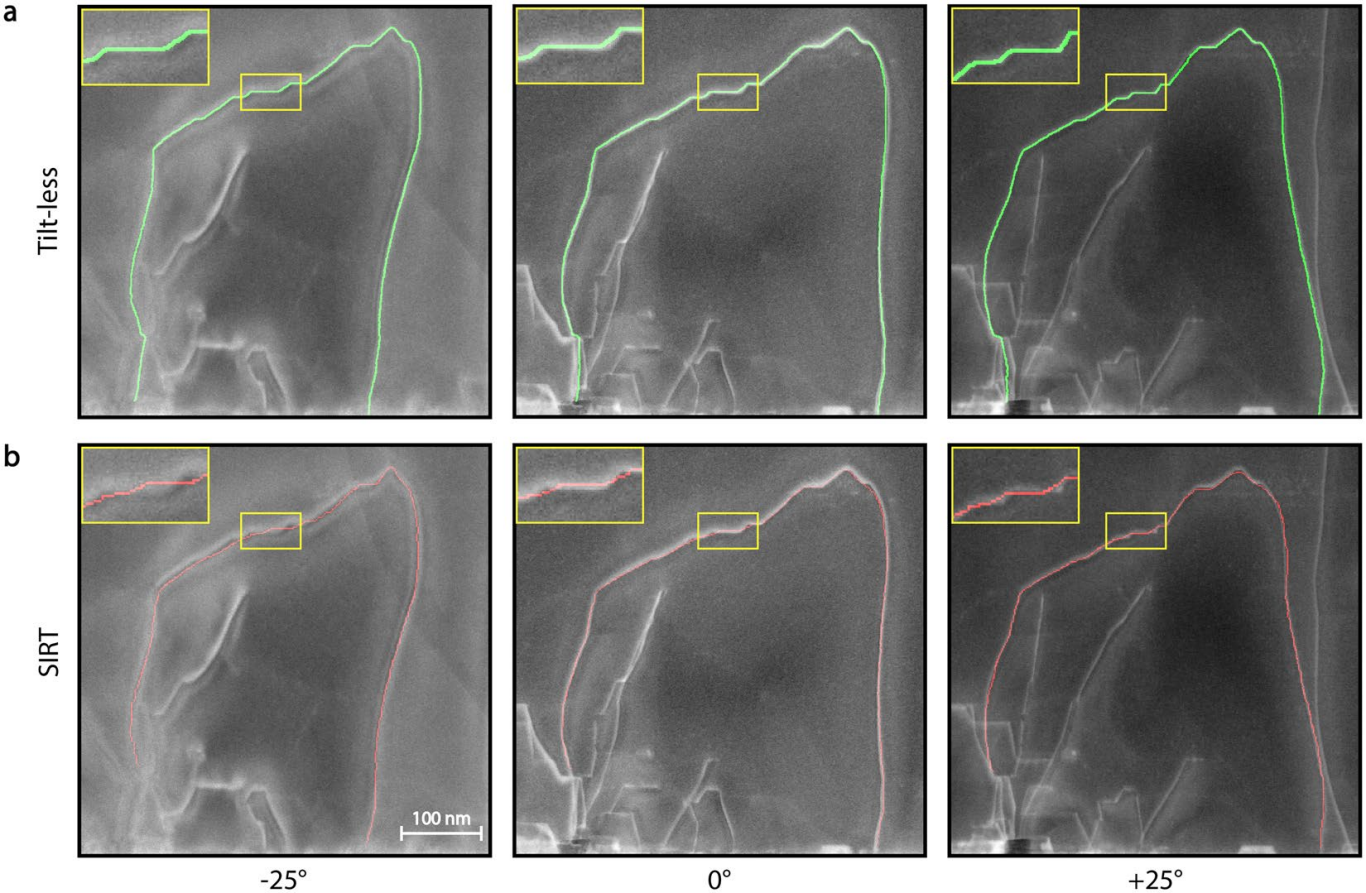
Comparison of the re-projections of a dislocation reconstructed in 3-D using the tilt-less and electron tomography techniques. Reconstructions are re-projected onto the micrographs that were acquired at different mechanical tilt angles. (**a**) Tilt-less STEM (of ±13.6 mrad virtual tilt). (**b**) STEM tomography using the SIRT algorithm (tilt-series from −25° to +25° at 1° increments).

The root-mean-square distance (RMSD) between the respective sets of dislocation paths identified between the tilt-series/SIRT and tilt-less techniques is 5.8 nm, indicating a good match between both approaches. To evaluate the accuracy of the reconstructions, 3-D dislocation paths obtained using the two methods are re-projected onto the micrographs acquired at different tilt angles (Figure 4). Although obtained from a single sample orientation, the tilt-less technique yields re-projections that closely match the experimental dislocation image, whereas those of the SIRT deviate slightly from their original 2-D contours. The main reasons for these minor differences are the artefacts associated with the low tilt range used for the STEM tomography (resulting from specimen and mechanical constraints), as well as the changes in the dislocation width due to diffraction conditions varying with the specimen tilt. Both techniques are subject to specific limitations. In the case of the tilt-series tomography this is the missing wedge effect, which essentially limits resolution on the *Z*-axis (thickness axis of the sample). Taking the definition of resolution as being the smallest distance between two points such that they can be resolved, the tilt-less technique has a *Z*-axis resolution defined by the minimum distance required to discriminate two objects superposed along the *Z*-axis. This is different to the *Z*-axis reconstruction accuracy assessed earlier, since that referred to the accuracy of locating an already discriminated object. To assess this *Z*-axis resolution, we start by discussing the hypothetical case of two perfectly straight dislocations, exactly superposed on the (*X*, *Y*) axes, and identify the minimum distance separating them on the *Z*-axis that would be needed to discriminate them. Taking a simple geometrical consideration^61^, when projected with a viewing angle equal to our tilt angle, the two dislocations must appear separated by a distance at most equal to the dislocation’s width. Keeping in mind that the line width of a dislocation image varies depending on diffraction condition and the dislocation’s position in the specimen, assuming a realistic line width of 2 nm and taking a tilt angle (convergence semi-angle) of 13.6 mrad leads to a *Z*-axis depth-resolution of 73 nm. Note however that this corresponds to a worst-case scenario, given the underlying assumption that the two dislocations are both perfectly straight and parallel. In contrast, real dislocations tend to take wriggling paths and will usually cross each other on the *Z*-axis at an angle to each other. Given the way our algorithm functions, integrating advanced computer vision methodologies to accurately detect the paths and match them to images, these factors will help discriminate *Z*-axis superposed dislocations, thereby improving effective *Z*-resolution compared to this estimate. Note that the algorithm also positions the dislocation line more accurately than its image contrast line width. As a result, we can resolve a complicated dislocation network with *Z*-axis superposed dislocations, as shown in Figure 3a and Supplementary Movies. Overall, noting as well that the *Z*-axis accuracy for positioning a discriminated dislocation is also different and superior to the *Z-*resolution, these results demonstrate that our tilt-less technique yields rapid and straightforward reconstructions equivalent to, or even more reliable than, those of standard electron tomography.

## Discussion

Using the example of dislocations, we have experimentally demonstrated and validated a tilt-less electron tomography technique for 3-D imaging of complex curvilinear nan﻿ostructures. Compared to the standard tilt-series electron tomography, this technique offers two key advantages. Firstly, data are required much faster (a few minutes of microscope/sample alignment and acquisition of 2 micrographs compared to >1 h for acquiring ~100 micrographs), with corresponding significant reduction in specimen exposure to the electron beam. Indeed, the total electron beam dose can be two orders of magnitude lower than for conventional tilt-based tomography. Secondly, its immunity to complications arising from mechanical tilt: While in the previous section this was discussed in the context of imaging dislocations, and will in particular help with 3-D reconstruction of dislocations in polycrystalline or elastically anisotropic materials, the ability to record a stereo pair of images under identical diffraction conditions is generally applicable to any diffraction contrast imaging. By eliminating mechanical lateral shift between the images, experimentally dependent need for fiducial alignment markers is also removed.

In terms of electron optical alignment, it suffices to focus and correct for astigmatism on just one STEM image in the tilt-less pair as the symmetry of aberrations gives correct focus and astigmatism for the other image as well, leading to a precise 2-D line detection and reconstruction. This brings an additional advantage for 3-D imaging in magnetic materials (see Supplementary Figures S5 and S6), for which the varying magnetic field of the sample at different mechanical tilt angles produces focus and astigmatism changes that effectively prohibit successful tilt-series electron tomography^60^. Another benefit of the tilt-less imaging technique resides in the fact that reconstructions are indexed in the crystallographic coordinate system of the specimen. The approach therefore gives fundamental information needed for evaluating dislocation arrangements, such as slip plane, direction and segment length.

While it is demonstrated here with the 3-D characterization of dislocation arrangements, the tilt-less technique is applicable to the analysis of other curvilinear nanometric objects in materials research and the life sciences, for instance to resolve the 3-D shape of DNA molecules. Equally, the technique can be used to identify the distribution of zero dimensional objects such as atomic clusters or nanoparticles embedded within a matrix, and requires only a standard modern FEG transmission electron microscope equipped with scanning mode and bright-field or annular detectors. Looking forwards, the emergence of segmented STEM detectors gives the extra capability to acquire the pairs of stereo images simultaneously, as demonstrated in Supplementary Figure S7. As a next step, the angular precision of this simultaneous acquisition will be improved by implementing specially designed double-hole apertures in the BFP.

In summary, by adopting a principle of rotating the beam/detector rather than object, we have experimentally demonstrated a fast, reliable TEM method for obtaining 3-D structural information of 0-D and 1-D objects using a single specimen tilt. The technique is a breakthrough in the 3-D visualization of curvilinear features, by offering a data acquisition speed and electron dose reduction of almost two orders of magnitude compared to standard TEM tomography, as well as free control over the tilt axis for imaging of crystal defects in individual grains of polycrystalline structures. When combined with segmented STEM detectors to acquire the stereo image pair simultaneously, we anticipate that it will pave the way for real-time 3-D TEM observation of the evolution of curvilinear features in dynamic processes, for instance during *in-situ* experiments on dislocation motion.

## Methods

### Electron microscopy instrumentation

Tilt-less 3-D electron imaging was performed using an FEI Tecnai OSIRIS transmission electron microscope in scanning mode at an accelerating voltage of 200 kV. This microscope is equipped with a central bright-field (BF) and three annular detectors of different collection angles. During the experiments, the illumination convergence angle (2α) of electrons was set to 27.2 mrad, using a nominal 70 µm probe-forming (CTEM condenser) aperture in the nanoprobe mode. At this convergence angle, image blurring due to spherical aberration of the probe-forming lens is negligible. The pairs of stereo BF-STEM images of 2048 × 2048 pixel resolution were acquired by selecting a camera length of 1.1 m such that the two edges of the (0 0 0) disc partially illuminated the nominal high-angle annular dark-field (HAADF) detector, which was hence effectively used as an annular BF detector. The two images are differentiated by inserting an objective aperture of ~10 mrad diameter to select either beam 1 or 2 (see Figure 2). In the absence of this blocking BFP aperture, the BF and HAADF detectors at the selected camera length have angular collection ranges of 0 to 3.7 mrad and 6.8 to 41.8 mrad, respectively.

For simultaneous acquisition of stereo images (Supplementary Figure S7), a double Cs-corrected FEI Titan Themis 60–300 equipped with a segmented annular dark-field detector was used. During the experiments, the illumination convergence angle (2α) of electrons was set to 42 mrad and images were acquired at 200 kV accelerating voltage and a 115 mm camera length such that the edges of the (0 0 0) disc illuminated two opposite channels of a four quadrants “DF4” segmented STEM detector.

### Specimen preparation

The InAlN/GaN hetrostructure was grown in an AIXTRON 200/4 RF-S metal organic vapour-phase-epitaxy (MOVPE) system on ***c***-plane sapphire substrate. Details on the sample growth can be found elsewhere^62^. For STEM observations a cross-sectional specimen with $[1\,\bar{1}\,0\,0]$ foil direction was prepared using a conventional focused ion beam (FIB) lift-out technique in a Zeiss NVision40 FIB/SEM workstation. The lamella, containing both GaN buffer layer and sapphire substrate, was extracted using a Kleindiek^TM^ micromanipulator, then attached to a copper Omniprobe^TM^ grid, and thinned down to a final thickness of ~ 250 nm. Final low-energy Ga^+^ polishing was performed at 2 kV and 60 pA.

The investigated ferritic steel sample was a thin foil of Fe-10Cr (wt. %) alloy, with a grain size between 50 and 300 µm and dislocation density <10^8^ cm^−2^. The TEM specimen was prepared by electro-polishing at −20 °C and 20 V using an 11% solution of perchloric acid in ethanol with the addition of 2% of butoxyethanol. Details on sample preparation can be found elsewhere^63^.

### Synthetic reconstruction of the model object

The fidelity of the reconstruction algorithm was evaluated on the reconstruction of the 3-D path of a model curvilinear object. From this model object a tilt-series of synthetic projections was generated (with different resolutions: 512 × 512, 1024 × 1024, and 2048 × 2048 pixels) with an increment of 0.1°. Different sets of reconstructions were made using an ensemble of only three images within different tilt ranges: from ±0.1° to ±25°. For each reconstruction, the root-mean-square distance (RMSD) and maximum error between the equivalent points of the model and reconstructed shapes were measured. This operation was performed on tilt-series of different pixel sizes (same object size but described in images of 512, 1024, and 2048 pixel width), in order to evaluate the effect of pixel size on the error.

### Anaglyph making

The anaglyphs were constructed by ﻿combi﻿ning differently filtered coloured﻿ stereo images﻿, one for each eye: the red colour component is removed from the image for the right eye, and﻿ the left eye image is filtered to remove the blue and green components.

## Electronic supplementary material

Supplementary Information

Movie1

Movie2

Movie3
