# Supplementary material for: Tilt-less 3-D electron imaging and reconstruction of complex curvilinear structures
Source: Sci Rep. 2017 Sep 6;7:10630. doi: 10.1038/s41598-017-07537-6 (PMC5587565; doi:10.1038/s41598-017-07537-6)
Supplement: Supplementary file 1 — Supplementary Information [file 41598_2017_7537_MOESM1_ESM.pdf]

Tilt-less 3-D electron imaging and reconstruction of complex curvilinear structures

Supplementary Materials

Emad Oveisi[1,2,*], Antoine Letouzey[2,3], Duncan T.L. Alexander[1], Quentin Jeangros[1]

Robin Schäublin[4], Guillaume Lucas[2], Pascal Fua[3] and Cécile Hébert[1,2,*]

[1] *Interdisciplinary Centre for Electron Microscopy*

École Polytechnique Fédérale de Lausanne (EPFL), CH-1015 Lausanne, Switzerland

[2] *Electron Spectrometry and Microscopy Laboratory*

École Polytechnique Fédérale de Lausanne (EPFL), CH-1015 Lausanne, Switzerland

[3] *Computer Vision Laboratory*

École Polytechnique Fédérale de Lausanne (EPFL), CH-105 Lausanne, Switzerland

[4] *Scientific Centre for Optical and Electron Microscopy*

Swiss Federal Institute of Technology in Zurich (ETHZ), CH-8093 Zurich, Switzerland

* Corresponding authors:
EPFL, MXC-135, Station 12, CH-1015 Lausanne, Switzerland; emad.oveisi@epfl.ch
EPFL, MXC-131, Station 12, CH-1015 Lausanne, Switzerland; cecile.hebert@epfl.ch

Contents:

Supplementary Figures

- Figure S1 ………………………………………………………………………………………….. 3
- Figure S2 ………………………………………………………………………………………….. 5
- Figure S3 ………………………………………………………………………………………….. 6
- Figure S4 ………………………………………………………………………………………….. 7
- Figure S5 ………………………………………………………………………………………….. 8
- Figure S6 ………………………………………………………………………………………….. 9
- Figure S7 ………………………………………………………………………………………... 10

Supplementary Movies

- Movie 1 ……………………………………………………………………………………………. 11
- Movie 2 ……………………………………………………………………………………………. 11
- Movie 3 ……………………………………………………………………………………………. 11

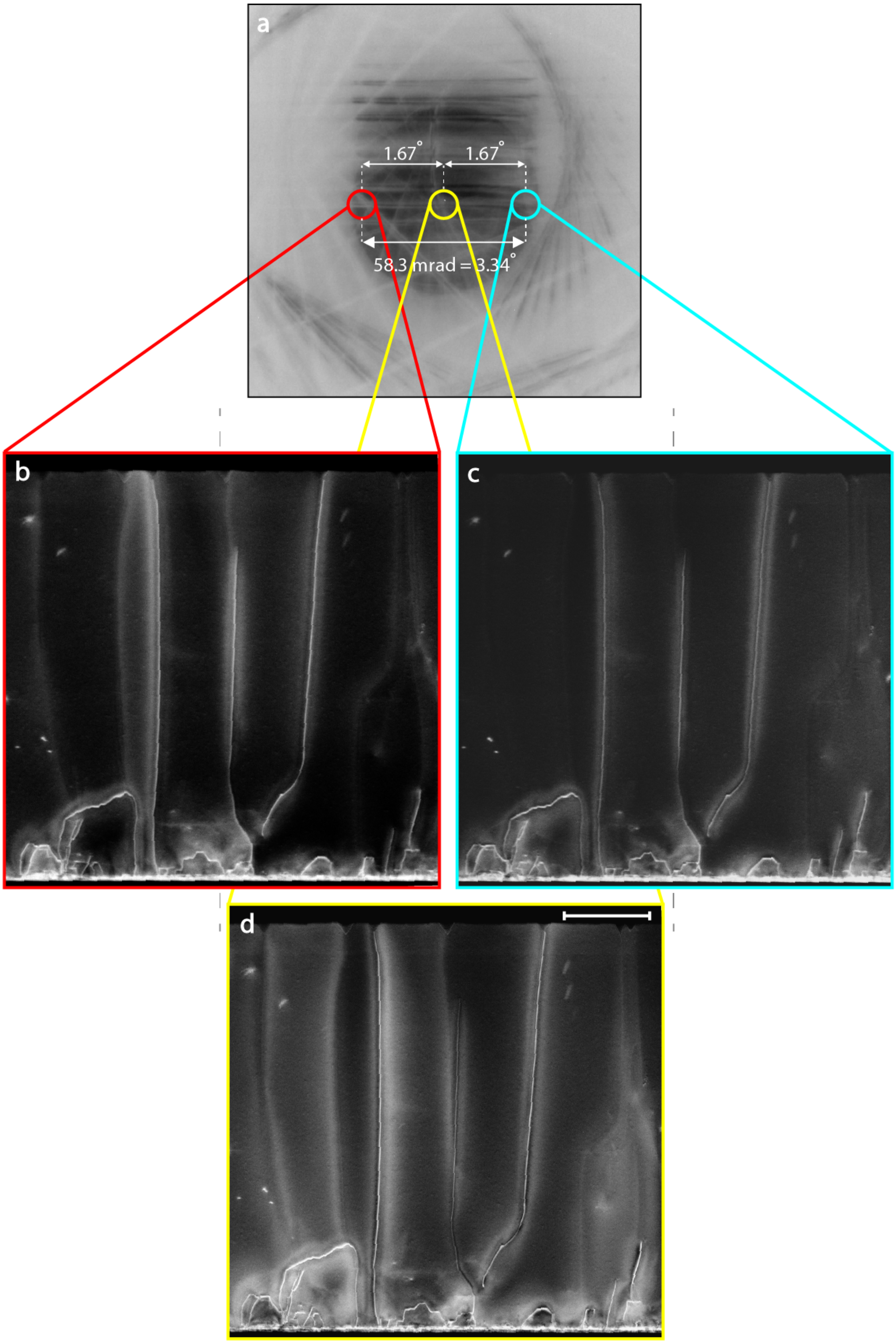

Figure S1. Tilt-less STEM imaging of dislocations of Figure 2 using a larger beam convergence angle of 58.3 mrad. (**a**) Illustration of the stationary CBED pattern of the

specimen (58.3 mrad beam convergence angle) set with positive deviation from a $\boldsymbol{g}_{(h\ k\ l)}$=(0 0 2) two-beam Bragg diffraction condition. (**b-d**) The BF-STEM stereo micrographs (inverted contrast) with a virtual tilt angle of 29.1 mrad (1.67°) between them. Scale bar, 500 nm.

Compared to Figure 2, the increase in convergence angle has two main consequences. Firstly, the virtual tilt angle of the stereographic pair used for reconstruction increases. This can improve the depth resolution of the reconstruction, as long as the increased aberrations associated with the larger convergence angle do not create artefacts in the images. Indeed, in this case the final reconstruction quality was equivalent to the case in Figure 2. Secondly, the on-axis image **d** consists of intensity contributions from both the (0 0 0) disc and an overlapping part of the (0 0 2) disc. However, even with this overlap image **d** shows that the dislocations remain well defined. This is reasonable because the rays of the (0 0 2) disc contributing to this image necessarily have a large deviation parameter and so will make much weaker contributions to the overall image intensity than the direct beam rays.

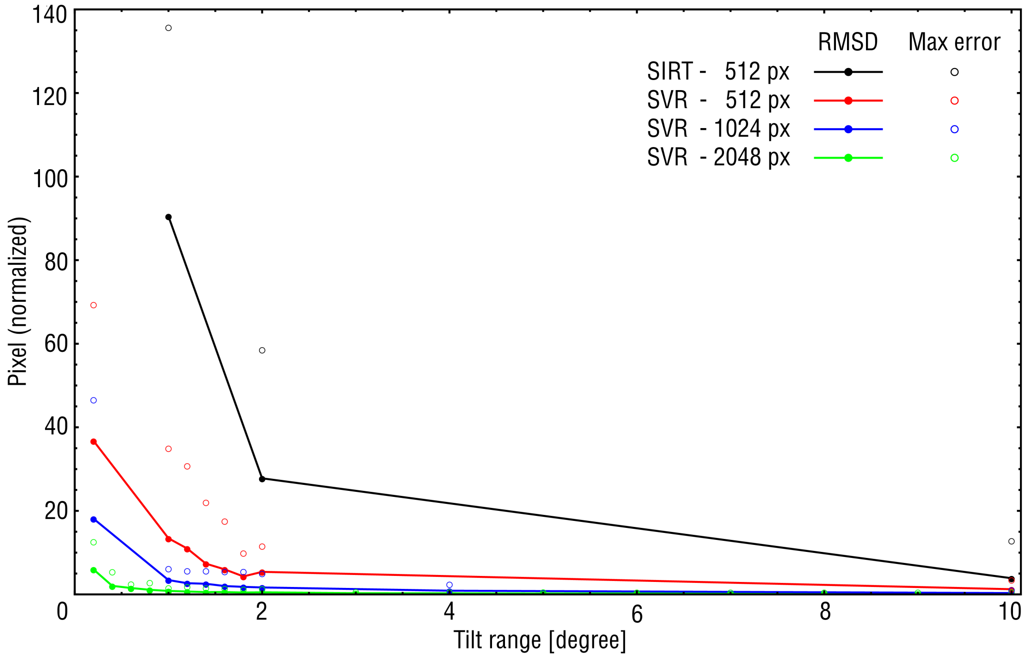

Figure S2. Illustration of reconstruction accuracy. Comparison of fidelity between the stereovision reconstruction algorithm (SVR, presented in this paper) and SIRT for the reconstruction of the 3-D path of a model curvilinear object from its synthetic projections. Lines and empty circles respectively indicate the root-mean-square distance (RMSD) and maximum error between the model and reconstructed shapes. Note that, in order to allow a direct comparison of the errors, the RMSD and maximum error of the 1024 and 2048 pixel width images are normalized to their corresponding error if they were to be reduced to a 512 pixel width image.

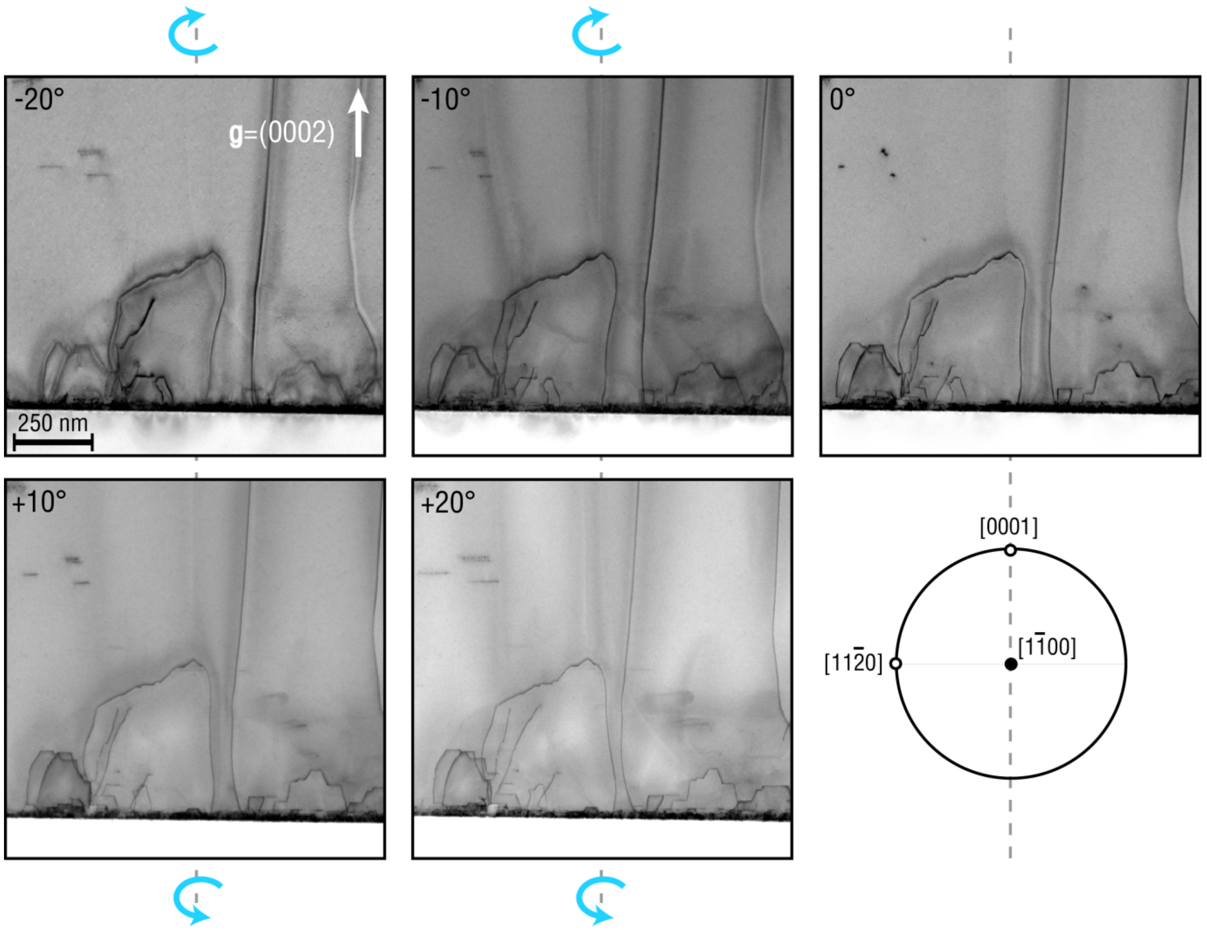

Figure S3. Tilt-series of BF-STEM images of dislocation arrays of Figure 2. Micrographs show at 10° interval dislocations in a ~ 250 nm thick specimen with a $[1\,\bar{1}\,0\,0]$ foil direction, extracted from the GaN layer of an ultra-thin InAlN/GaN heterostructure. The images are shown with the [0 0 0 1] growth direction pointing vertically upward. The tilt-series was acquired at 200 kV under two beam diffraction condition with $\boldsymbol{g}_{(h\,k\,l)}$= (0 0 2) within ±25° angular range. Dashed lines show the tilt axis. Crystallographic orientation of the specimen as well as the tilt axis is shown with a stereographic projection in the lower part of the figure.

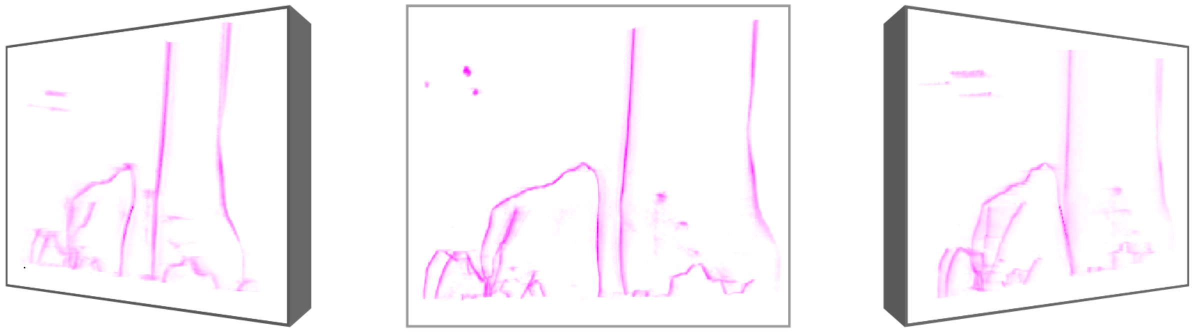

Figure S4. Tomographic reconstruction (SIRT) of dislocation arrays of Figure 2. The reconstruction was made from the tilt-series shown in Figure S3. More details on the reconstruction in the text.

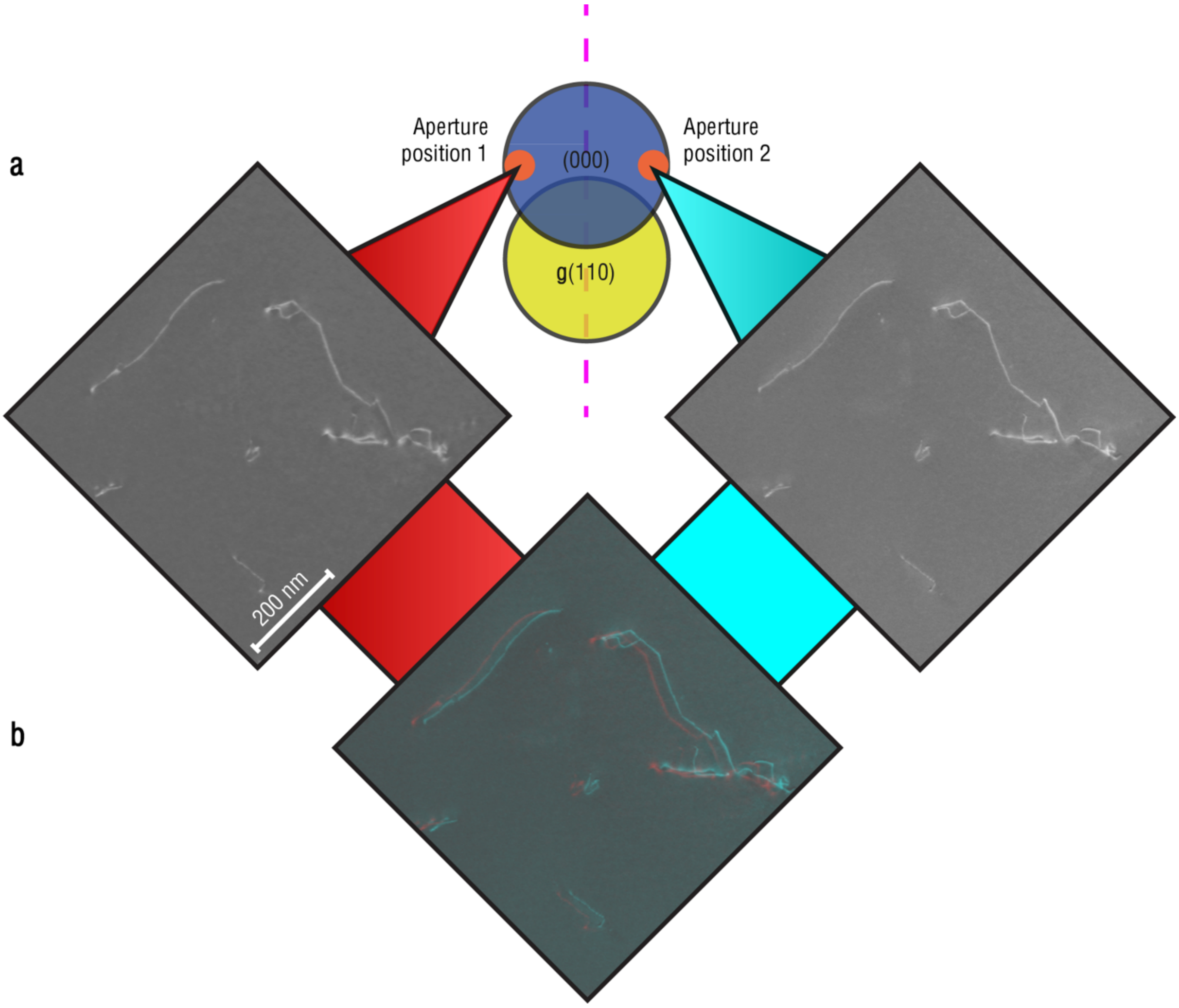

Figure S5. Tilt-less electron tomography of dislocations in a ferritic steel alloy. The schematic illustrates a CBED pattern in $\boldsymbol{g}_{(h\ k\ l)}$= (1 1 0) two-beam diffraction condition. (**a**) BF-STEM stereo micrographs belonging to BFP aperture positions 1 and 2, respectively. Image contrast is inverted for a better visibility. (**b**) Corresponding anaglyph.

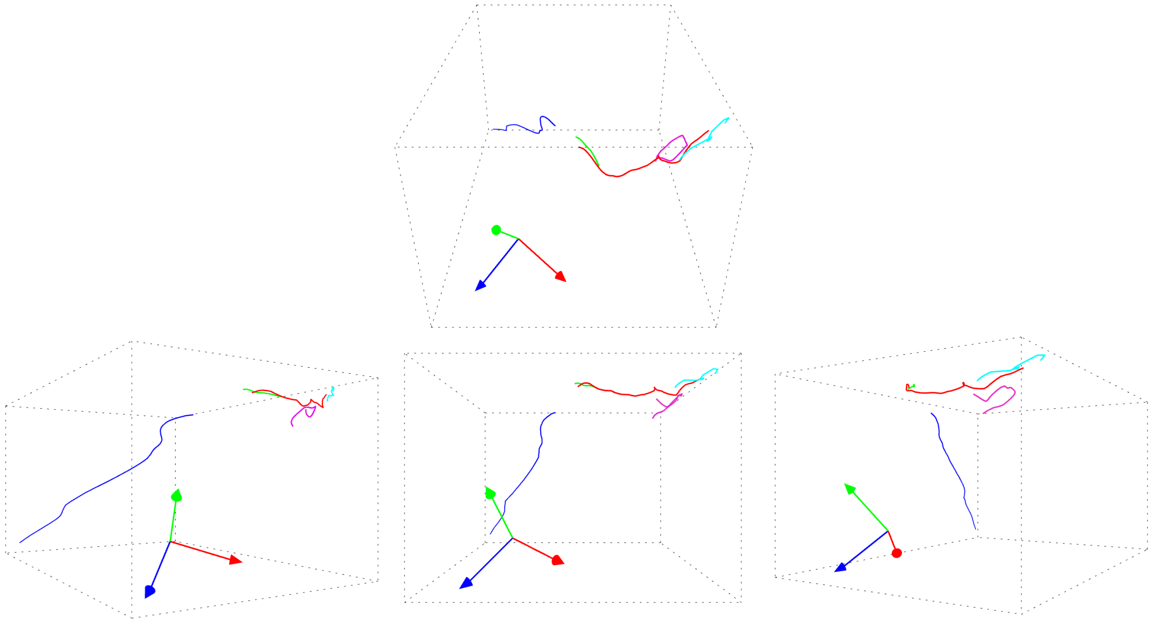

Figure S6. Tilt-less 3-D reconstruction of dislocations of Figure S5. The green, blue, and red arrows correspond to the principal crystallographic [1 0 0], [0 1 0], and [0 0 1] axes, respectively. The central reconstruction is viewed along the same direction as in the microscope. Note that reconstructions are represented in perspective view and thus may appear different from the corresponding micrographs in Figure S5 (orthographic projections).

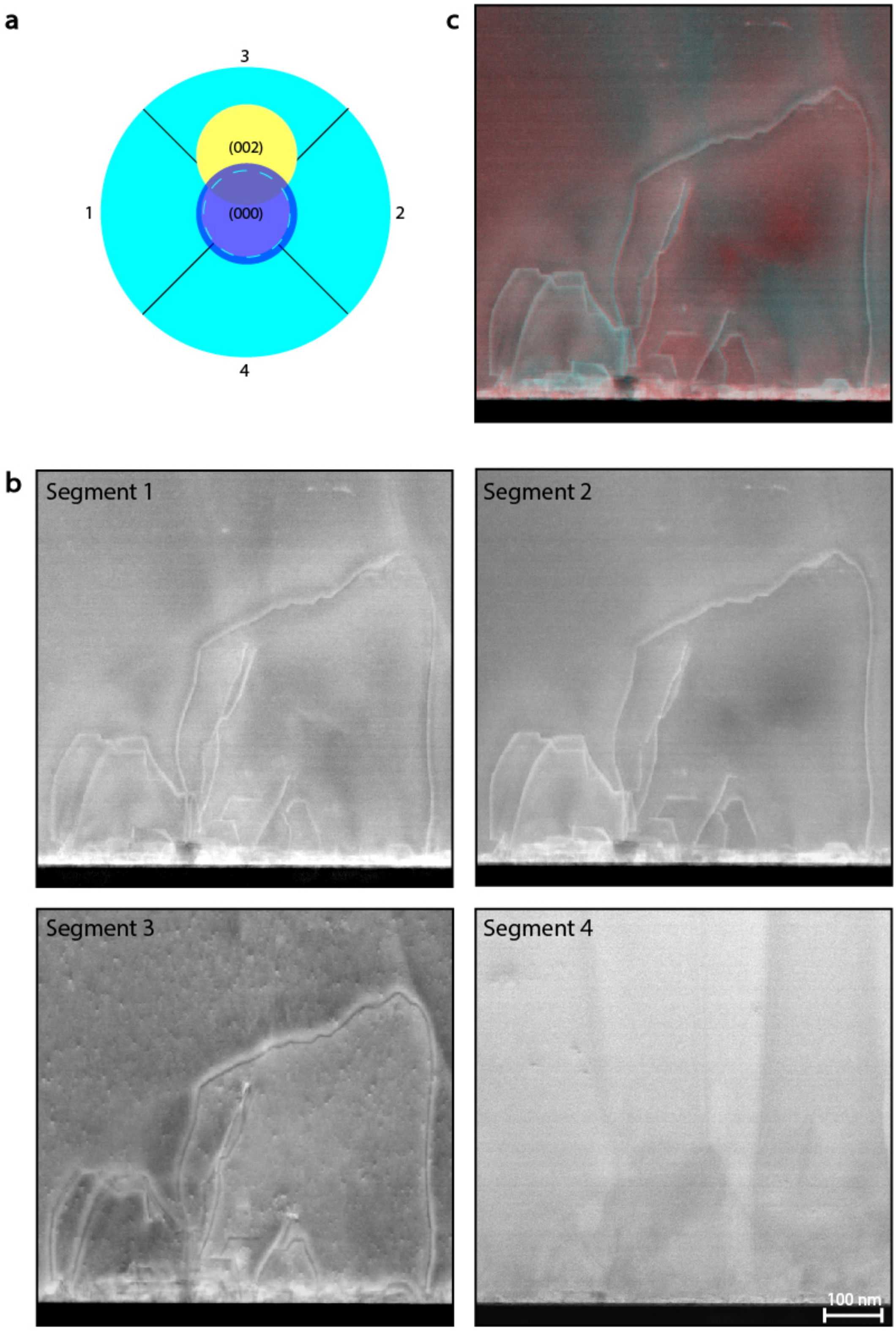

Figure S7. Single-acquisition 3-D electron imaging using segmented STEM detector. (**a**) Schematic illustration of the CBED pattern and segmented STEM detector. Dashed circle indicates the inner part of the detector. Drawings are not to scale.

(**b**) BF-STEM micrographs (inverted contrast) of dislocations in the GaN layer of an InAlN/GaN-based heteroeptaxial layer, obtained using different channels of a four quadrants "DF4" segmented STEM detector. Edges of the (0 0 0) disc of same deviation parameter (perpendicular to the diffraction vector $\boldsymbol{g}_{(h\ k\ l)}$=(0 0 2)) illuminate two opposite channels (channels 1 and 2) of the detector, giving, as expected, images analogues to those obtained by moving the BFP aperture perpendicular to the diffraction vector (see Figure 2 in the main text). Conversely, channels 3 and 4 of the segmented STEM detector overlap opposite edges of the (0 0 0) along the diffraction vector, giving images with different diffraction contrasts. (**c**) Anaglyph illustrating the 3-D configuration of dislocations, produced by combining the stereo micrographs that were acquired using channels 1 and 2.

Supplementary Movies

Movie 1. Tilt-less 3-D reconstruction of dislocation arrays of Figure 2.

Movie 2. BF-STEM tomography tilt-series of dislocation arrays of Figure 2.

Movie 3. Tomographic (SIRT) reconstruction of dislocation arrays of Figure 2.
